# Dual Energy X-ray Absorptiometry (DEXA) as a longitudinal outcome measure of cancer-related muscle wasting in mice

**DOI:** 10.1371/journal.pone.0230695

**Published:** 2020-06-19

**Authors:** Calvin L. Cole, Christopher A. Beck, Deja Robinson, Jian Ye, Bradley Mills, Scott A. Gerber, Edward M. Schwarz, David Linehan

**Affiliations:** 1 Department of Orthopaedics, University of Rochester Medical Center, Rochester, New York, United States of America; 2 Center for Musculoskeletal Research, University of Rochester Medical Center, Rochester, New York, United States of America; 3 Department of Surgery, University of Rochester Medical Center, Rochester, New York, United States of America; 4 Cancer Control, University of Rochester Medical Center, Rochester, New York, United States of America; 5 Dept of Biostatistics and Computational Biology and the Dept of Orthpaedics; 6 Department of Microbiology, University of Rochester Medical Center, Rochester, New York, United States of America; 7 Department of Radiation Oncology, University of Rochester Medical Center, Rochester, New York, United States of America; 8 Wilmot Cancer Institute, University of Rochester Medical Center, Rochester, New York, United States of America; Indiana University School of Medicine, UNITED STATES

## Abstract

**Introduction:**

Pancreatic ductal adenocarcinoma (PDAC) is notorious for its associated skeletal muscle wasting (SMW) and mortality. Currently, the relationships between PDAC, SMW, and survival are poorly understood. Thus, there is great need for a faithful small animal model with quantitative longitudinal outcome measures that recapitulate clinical PDAC, to define SMW onset and assess progression. Therefore, we aimed to validate dual energy X-ray absorptiometry (DEXA) as a longitudinal measure of lean mass, and demonstrate its utility to quantify SMW in the KCKO murine model of PDAC.

**Methods:**

In vivo body composition of: 1) untreated mice at 5, 8, 12, 18, and 22 weeks of age (n = 4) and 2) a cohort of mice with (n = 5) and without PDAC (n = 5), was determined via DEXA and lean mass of the lower hind limbs was predicted via a region of interest analysis by two-independent observers. Total body weight was determined. Tibialis anterior (TA) muscles were weighed and processed for histomorphometry immediately post-mortem. Statistical differences between groups were assessed using ANOVA and Student’s t-tests. Linear regression models and correlation analysis were used to measure the association between TA and DEXA mass, and reproducibility of DEXA was quantified via the intraclass correlation coefficient (ICC).

**Results:**

Lean mass in growing untreated mice determined by DEXA correlated with TA mass (r^2^ = 0.94; p <0.0001) and body weight (r^2^ = 0.89; p <0.0001). DEXA measurements were highly reproducible between observers (ICC = 0.95; 95% CI: 0.89–0.98). DEXA and TA mass also correlated in the PDAC cohort (r^2^ = 0.76; p <0.0001). Significant SMW in tumor-bearing mice was detected within 38 days of implantation, by DEXA, TA mass, and histomorphometry.

**Conclusions:**

DEXA is a longitudinal outcome measure of lean mass in mice. The KCKO syngeneic model is a *bona fide* model of PDAC associated SMW that can be quantified with longitudinal DEXA.

## Introduction

Pancreatic ductal adenocarcinoma (PDAC) is the most common malignancy of the pancreas, and is the fourth leading cause of cancer-related deaths, with its incidence expected to increase over the coming decade [1, 2]. Despite advances in the treatment of PDAC, its 5-year survival rate stands at 9% [2]. Additionally, treatment intolerance and/or discontinuation of treatment, continue to present challenges for patients with PDAC and their caregivers. Most notable among the detractors of quality of life for PDAC patients is sarcopenia, also known as skeletal muscle wasting (SMW), which is a growing burden among cancer survivors [3, 4]. As such, SMW is prognostic of treatment failure, radiotherapy toxicity, and a shorter time to tumor progression related to survival [4–7]. SMW is a progressive and generalized skeletal muscle disorder that is associated with increased likelihood of adverse outcomes including falls, fractures, physical disability, poor quality of life and mortality [8]. Importantly, a large percentage of patients with PDAC experience cancer-related SMW and these patients have reduced physical function, increased postoperative morbidity, reduced response to treatment, and shorter life expectancy [9]. Furthermore, SMW has been identified as a prognostic factor in pancreatic cancer [10] and is an independent predictor of infectious disease and postoperative mortality in resected patients [11, 12]^,^. Thus, reductions in the incidence of SMW in patients with pancreatic cancer may reduce disease and treatment-related complications, which adversely affect dose and length of treatment.

Currently, the time of onset of SMW following a cancer diagnosis is poorly understood. However, clinical studies suggest that once cancer-related SMW is initiated, it is irreversible [13]. Therefore, a priority in the treatment of PDAC-related muscle wasting must be determining when it is initiated and preventing its establishment. The anatomical distance between tumor cells and sites of SMW posit that inflammatory cytokines may transmit systemic signals that potentiate muscle wasting through the alteration of myofibrillar intracellular pathways regulated by both hormones and cytokines that slow protein synthesis and accelerate catabolism [9, 14]. Unfortunately, further elucidations of PDAC-related SMW and identification of treatable targets have been challenging due to the absence of small animal models with longitudinal outcomes. Thus, the development of preclinical PDAC models that recapitulate clinical PDAC-related muscle wasting are needed to investigate paracrine factors that may be emitted in the early stages of a cancer diagnosis and perpetuate SMW.

Early recognition of SMW may also aid clinicians in devising an appropriate dosing algorithm to reduce treatment toxicity while improving treatment tolerance and related outcomes of the cancer diagnosis. In the context of non-metastatic disease, the only possible cure for PDAC is surgical resection [15]. However, less than 20% of PDAC patients meet the criteria for resection due to the locally advanced or metastatic nature of their diagnosis [6]. Recent research suggests that neoadjuvant treatment (NAT) may help to improve the resectability rates among patients with PDAC but, may have an adverse effect on body composition that worsen post-surgical outcomes or reduce resection opportunities [6]. Among many other reasons, the adverse effect of NAT may be the result of an incorrect dosing regimen that is based on body weight or body mass index (BMI). Indeed, studies have shown the measurement of lean mass to be a more superior indicator of treatment toxicity and dosing response in patients who experience cancer-related SMW, when compared to body weight and BMI [16]. Therefore, it may be beneficial to monitor a patient’s lean mass before, during, and after NAT to determine the early need for additional intervention. In a homeostatic myocellular environment, pathways that regulate protein synthesis and breakdown function to prevent unnecessary protein cycling. However, in an environment of SMW, a dysregulation of anabolic and catabolic systems exists that results in a net loss of protein. A determination of the timing of this dysfunction may play a key role in understanding the mechanism(s) that lead to PDAC-related SMW.

Dual-energy X-ray absorptiometry (DEXA) has emerged as a viable, non-invasive method of serial in vivo body composition analysis in small animals due to its feasibility, accuracy, and reproducibility [17]. Additionally, DEXA is widely used for body composition measurements in humans for both clinical and research purposes [8]. In fact, an article on the European consensus definition of Sarcopenia named DEXA as one of the most widely used tools to measure sarcopenia [8]. The fact that it is highly accessible, its results are highly reproducible, and it is very cost effective, are some of the reasons given for its popularity. In terms of its comparability to CT. Bredella et al [18] found strong correlations between DEXA- and CT-derived body composition measurements in anorexic, obese, and lean controls. Also, Guglielmi et al [19] state, “Due to DXA’s favorability in terms of accuracy, simplicity, availability, low cost and low radiation exposure, its role in sarcopenia diagnosis is becoming increasingly important, emerging as a reference assessment technique in muscle mass evaluation.” In regards to the comparability of DEXA and EchoMRI, Galgani et al found both techniques to be equally affective [20]. Other research also supports this finding [21]. Thus, DEXA outcomes in an animal model of disease may recapitulate clinical outcomes. Furthermore, use of a non-invasive form of lean mass (LM) measurement is vital during PDAC-related SMW to better understand the onset of this phenomenon. Indeed, researchers have been successful in using tools such as nuclear magnetic resonance (NMR) [22–24] and micro-computed tomography (micro-CT) [25] to longitudinally measure lean mass in various preclinical models of cancer cachexia. Therefore, we aimed to validate DEXA, as an additional resource for analyzing in vivo lean mass and demonstrate its utility to quantify SMW in a novel model of PDAC-related SMW [26]. Additionally, given DEXA’s broad utility in the, we believe its translational potential makes it a very attractive outcome measure for preclinical research.

To examine PDAC-related SMW, we used a Muc 1-null PDA model (designated KCKO). We chose this model because it lacks the *Muc*1 gene. Research shows that lack of Muc1 significantly decreases proliferation, invasion, and mitotic rates both in vivo and in vitro, when compared to PDAC cells containing Muc1 (designated KC and KCM) [27]. Furthermore, in the absence of Muc1, the pancreatic tumor burden and secondary metastasis are decreased. This feature, provides KCKO tumor-bearing mice a significant survival benefit compared to KC and KCM mice [27]. Research from our lab showed that KCKO tumor-bearing mice live more than 5 weeks longer than KC tumor-bearing mice (unpublished data). This extended survival benefit of the KCKO model provides an enhanced opportunity for therapeutic intervention before, during, and after the onset of PDAC-related SMW, which does not exist in other murine models of PDAC.

## Materials and methods

### Aged mice

The University of Rochester Medical Center University Committee on Animal Resources (UCAR) has approved of all animal work conducted herein. Female C57BL/6 mice were purchased from the Jackson Laboratory, Bar Harbor, ME (stock number 000664) at 4, 7, 11, 17, and 21 weeks of age. Mice were aged in a pathogen-free facility for one week, under an IACUC approved protocol. Briefly, on the day of sacrifice, mice (n = 20) were anesthetized using ketamine 100 mg/g of bodyweight. After sedation, mice underwent a full body DEXA scan. Mice were sacrificed via an overdose of ketamine and secondary cervical dislocation immediately after the DEXA scan and their Tibialis Anterior (TA) muscles were harvested and weighed for correlative analysis.

### Murine orthotopic model of pancreatic cancer

We utilized the murine syngeneic-orthotopic model of PDAC as previously described [27]. As there is no known sexual dimorphism in this model, only female C57BL/6J mice were used. They were obtained at 6–8 weeks of age from the Jackson Laboratory (stock number 000664) and maintained in a pathogen-free facility under an IACUC approved protocol. As the development of this model has been described elsewhere [27], we will only briefly describe the model. The murine PDAC cell line KCKO was a gift from the lab of Dr. David Linehan [26]. All cell lines tested negative for mycoplasma and were verified to be of C57BL/6 origin using CellCheck Plus (IDEXX BioResearch). The cells were maintained in complete Dulbecco's modified Eagle's medium (Invitrogen) supplemented with 10% FBS (HyClone), 1% glutamax (Invitrogen), and 1% penicillin/streptomycin. After a 1-week acclimation period, mice were randomized to one of two groups: PDAC (n = 5) or no tumor control (NTC) (n = 5). Mice in the PDAC group were anesthetized and injected in the tail of the pancreas with 2×10^5^ KCKO-luc cells suspended in a 1:1 PBS to Matrigel (Corning) mixture. NTC mice received no surgery and were sacrificed at a ratio of 1:1 with mice of the PDAC group. Mice were maintained in standard isolation cages with a 12hr light: dark cycle, and given ad libitum access to water and standard chow. Longitudinal DEXA scanning was performed on days 14, 35, 42, 49, and 56. The total length of this experiment was 56 days. Tumor-bearing mice were sacrificed when they developed end-stage disease, defined by exhibiting three or more characteristics of the Institutional Animal Care and Use Committee’s (IACUC) definition of “failure to thrive”. Characteristics of “failure to thrive” included, but were not limited to self-isolation, hunched over appearance, lack of or reduced cage activity, lack of or no resistance to scruffing, mangled hair appearance after scruffing, failure to eat or drink, and/or visual signs of breathing difficulty. To determine if these characteristics were exhibited, animals were checked twice daily, 30 days after tumor inoculation. This is the time point in which untreated animals reach an advanced stage of disease [26]. Mice in the non-tumor-bearing group were sacrificed concordantly to allow for intergroup comparisons. When animals were found to exhibit 3 or more characteristics of “failure to thrive” they were euthanized via ketamine over dose and secondary cervical dislocation. Following euthanasia, skeletal muscles (quadriceps, Extensor Digitorum Longus (EDL), Soleus (SOL), and TA) and PDAC tumors were harvested for histology, and cardiac puncture was performed to obtain serum for Luminex assay. Wet weight of TA muscles were taken for correlative analysis, before being flash frozen for histology.

### Dual-energy X-ray absorptiometry (DEXA)

Body composition was assessed in all mice using a DEXA scanner (PIXImus2; Lunar, Madison, WI). Mice were weighed before undergoing DEXA scan analysis. Each mouse was anesthetized for the duration of the procedure (5 min) with an i.p. injection of 100 mg/kg ketamine. Each mouse was placed on the scanner bed in the prone position, with the limbs and tail stretched away from the body. The PIXImus employs a cone beam X-ray source generating energies at 35 and 80 keV with a current of 0.5 mA for both energy levels. The detector is flat (100 × 80 mm) and comprised of individual pixels of 0.18 × 0.18 mm. Based on the attenuation of two energy levels, the system provides quantitative data on the fat tissue content, the lean tissue content, and the total tissue mass within the region of interest (ROI). One scan per mouse was performed and analyzed with PIXImus software (2.10; GE/Lunar). The head was excluded from calculation using a manual ROI. The PIXImus was calibrated with an aluminum/lucite phantom (corresponding to bone mineral density = 0.0592 g/cm2 and 12.5% fat) on each day of testing according to the manufacturer's instructions. Lean mass was calculated using the lower hindlimbs as an ROI to exclude the measurement of the tumor burden. Lean mass was calculated as an index of total mass minus fat mass using the following equation: total mass–((% fat x total mass)/100). For each mouse, lean mass was calculated for the lower right and left limb independently, and the average of both measurements were used as the final lean mass for the animal.

### Antibodies

The following antibodies were used: laminin (rat, 1:1500, Sigma-Aldrich, L0663) and DAPI (1:3000).

### Muscle histology

TA, SOL, and EDL muscles were harvested for histology. TA muscles were cut from the most distal and proximal TA tendon attachment, cleaned of extraneous tissue, blotted, and weighed. After which, TA, muscles were stored in a 30% mixture of sucrose in PBS for 24 hours, at which time, muscles were embedded in OCT (Tissue Tek), flash-frozen using dry ice and 2-methylbutane (Sigma-Aldrich), and processed for fresh-frozen histology as previously described [28]. 10μm sections were cut and stained with H&E and representative micrographs were obtained for descriptive analyses. Immunohistochemistry for laminin (extracellular matrix) and nuclei determination were also performed as previously described [28, 29], in which TA muscles were cryosectioned at 10 μm, to obtain transverse sections. Sections were permeabilized with PBS-T (0.2% Triton X-100 in PBS) for 10 min, blocked in 10% normal goat serum (NGS, Jackson ImmunoResearch) for 30 min at room temperature. If a mouse primary antibody was used, sections were blocked in 3% AffiniPure Fab fragment goat anti-mouse IgG (H+L) (Jackson ImmunoResearch) with 2% NGS in PBS at room temperature for 1 h. Primary antibody incubation was performed in 2% NGS/PBS at 4°C overnight. Secondary antibody incubation was carried out in 2% NGS/PBS at room temperature for 1 h. After washing in PBS, sections were counter-stained with DAPI to label myonuclei. All slides were mounted with Fluoromount-G (SouthernBiotech). Fluorescent microscopy was performed using a Zeiss Imager: M1m microscope with AxioVision SE64 software, and representative images were used to quantify muscle cell area.

### Fixed single fiber staining

Single myofiber size and myonuclear analysis was performed, as previously described [28]. For single myofiber size and myonuclear analysis, whole limbs (n = 19) were fixed in 4% PFA for 48 h prior to EDL and SOL muscle dissection. Fixed muscles were incubated in 40% NaOH for 2 h to induce dissociation, and single myofibers were gently titrated and washed in PBS prior to staining with DAPI. For quantification, the cross-sectional area (CSA) of 100 fibers per mouse was determined manually from digitally-photographed DAPI stained fibers at 100X, and averaged as the CSA for the mouse.

### Statistical analysis

Myofiber CSA was determined using ImageJ software. The diameter of the fiber was measured at three points along the fiber to get an average CSA. TA cell area was calculated by drawing an ROI around the extracellular space of 200 individual cells and taking the average of the sum of those cells. Results are presented as mean±sd. Statistical significance was determined using t-tests for single comparisons and one-way and two-way ANOVA for multiple comparisons. Pearson correlation coefficient was calculated to measure the association between DEXA whole body lean mass vs. DEXA lower limb lean mass, TA weight vs. DEXA lower limb lean mass, and body weight vs. DEXA lean mass. Linear regression models were used to evaluate DEXA lower limb mass as a predictor of DEXA Whole body lean mass, TA weight and body weight with and without adjustment for the effects of time (treated as a categorical covariate to avoid assuming linearity over time), with predictability measured by the model’s square root of mean squared error (rMSE). Reproducibility of DEXA was assessed via the intraclass correlation coefficient (ICC) along with its 95% CI. Analyses were performed using GraphPad Prism software (GraphPad Software, San Diego, CA, USA) version 7.2 and 8.0, R version 3.5.1, and SAS version 9.4. P<0.05 was considered significant (*P<0.05, **P<0.01, ***P<0.001, ****P<0.0001).

## Results

### In vivo DEXA measures are reliable and reproducible in predicting the body mass of growing mice

**Fig 1:**
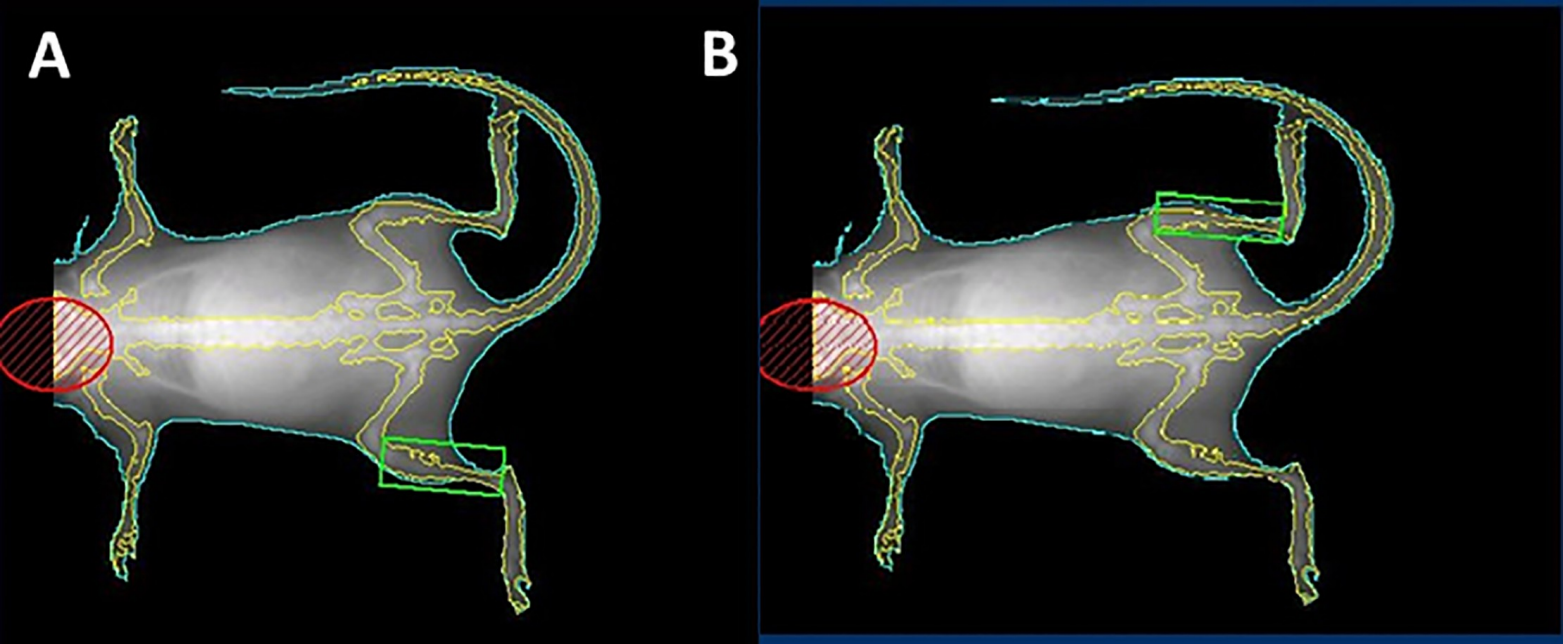
In vivo visualization and quantification of lean mass in a lower limb region of interest (ROI). A representative dual energy x-ray absorptiometry (DEXA) scan image of a mouse is shown to illustrate segmentation of the head (red oval), body (green outline), and the ROI for lean mass analysis (green box) of the (A) right and (B) left lower hind limbs as described in Material and Methods.

**Fig 2:**
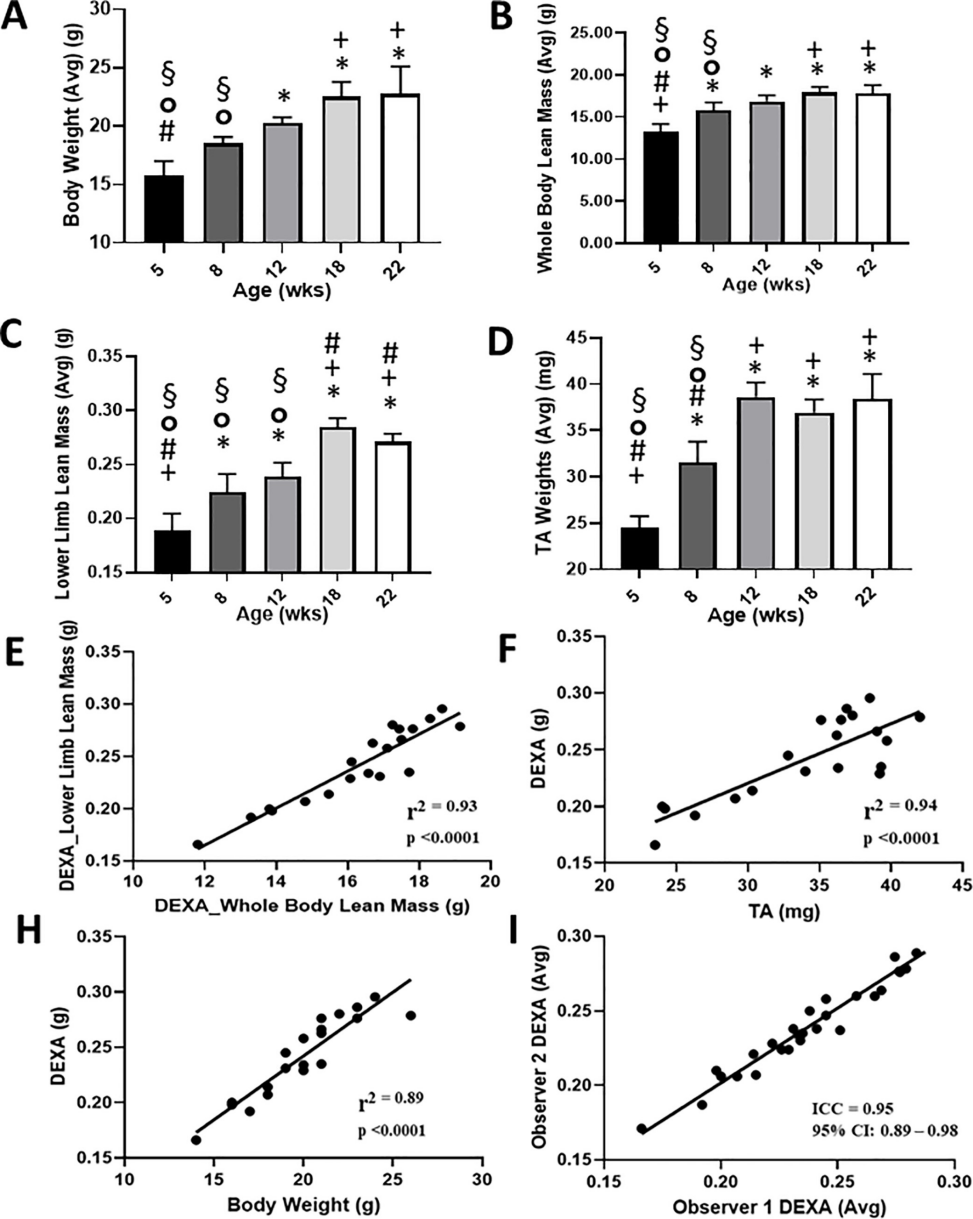
Validation of DEXA as an in vivo measure of lean mass in lower limbs of growing mice. Female C57BL/6 were weighed and DEXA scanned at 5, 8, 12, 18 and 22 weeks of age (n = 4 per group). Postmortem, mice Tibialis Anterior (TA) muscles were dissected and weighed immediately. One-way ANOVA with Tukey’s for multiple comparison test was used to determine the difference in (A) Bodyweight, (B) whole body lean mass (C) lower limb mass, and (D) TA weight for each mouse. All variables increased concomitant with age. Data (mean ±SD) were significantly different (p < 0.05): *versus 5 week, + versus 8 week, # versus12 week, ° versus 18 week, and § versus 22 week old mice. A Pearson correlation analysis was performed to determine the relationship between DEXA lean mass vs. (E) whole body lean mass, (F) TA weight, and (H) bodyweight. The analysis confirmed a significant (p < 0.0001 for whole body lean mass, TA weight, and bodyweight) direct correlation between the three measurements with r^2^ = 0.86, 0.94 and 0.89 for whole body lean mass, TA weight, and bodyweight, respectively. Data is expressed as mean ± SD. Lower limb lean mass was determined by DEXA at each time point by two independent observers as described in Fig 1. (I) The intraclass correlation coefficient (ICC = 0.95) shows a strong relationship between reader observations (95% CI: 0.89–0.98).

To assess the utility of longitudinal DEXA to assess in vivo murine body mass, we weighed and completed DEXA scans on 5, 8, 12, 18, and 22 week old mice (n = 4 per group) (Fig 1). Body weight was significantly different (5 weeks vs. 12**, 18****, and 22**** weeks) and (8 weeks vs. 18** and 22 weeks**) (**p<0.01, ****p<0.0001) (Fig 2A). DEXA revealed a significant increase in whole body mass ((5 weeks vs. 8**, 12***, 18**** and 22**** weeks); (8 weeks vs. 18* and 22* weeks)) and lower limb lean mass ((5 weeks vs. 8*, 12***, 18**** and 22**** weeks), (8 weeks vs. 18**** and 22** weeks), and (12 weeks vs. 18** and 22* weeks)) of growing mice (*p<0.05, **p<0.01, ***p<0.001, ****p<0.0001) (Fig 2B and 2C). TA weight analysis confirmed the DEXA results, its average also significantly increased with age in this cohort of growing mice ((5 weeks vs. 8***, 12****, 18**** and 22**** weeks), (8 weeks vs. 18**** and 22** weeks), and (12 weeks vs. 18** and 22* weeks)) (Fig 2D). In addition, regression modeling revealed substantial variation due to time, such that adding time as a covariate improved the predictability of lean mass by DEXA. Furthermore, when modeling DEXA whole body mass and TA weight using DEXA lower limb mass as a predictor, the analysis determined that DEXA lower limb mass has an rMSE of 0.59 g and 1.89 mg when predicting whole body mass and TA weights, respectively. Likewise, a Pearson correlation analysis demonstrated a strong relationship between DEXA lower limb mass and DEXA whole body mass (r^2^ = 0.93; p<0.0001) (Fig 2E), TA weight (r^2^ = 0.94; p<0.0001) and body weight (r^2^ = 0.89; p<0.0001) (Fig 2F–2H). Lower limb mass was measured by two independent observers and the reproducibility of DEXA proved to be excellent. The intra-class correlation coefficient (ICC) analysis completed on these measures shows strong agreement between the two separate observations (ICC = 0.95; 95% CI: 0.89–0.98) (Fig 2I).

### DEXA predicts skeletal muscle wasting in a murine model of PDAC

**Fig 3:**
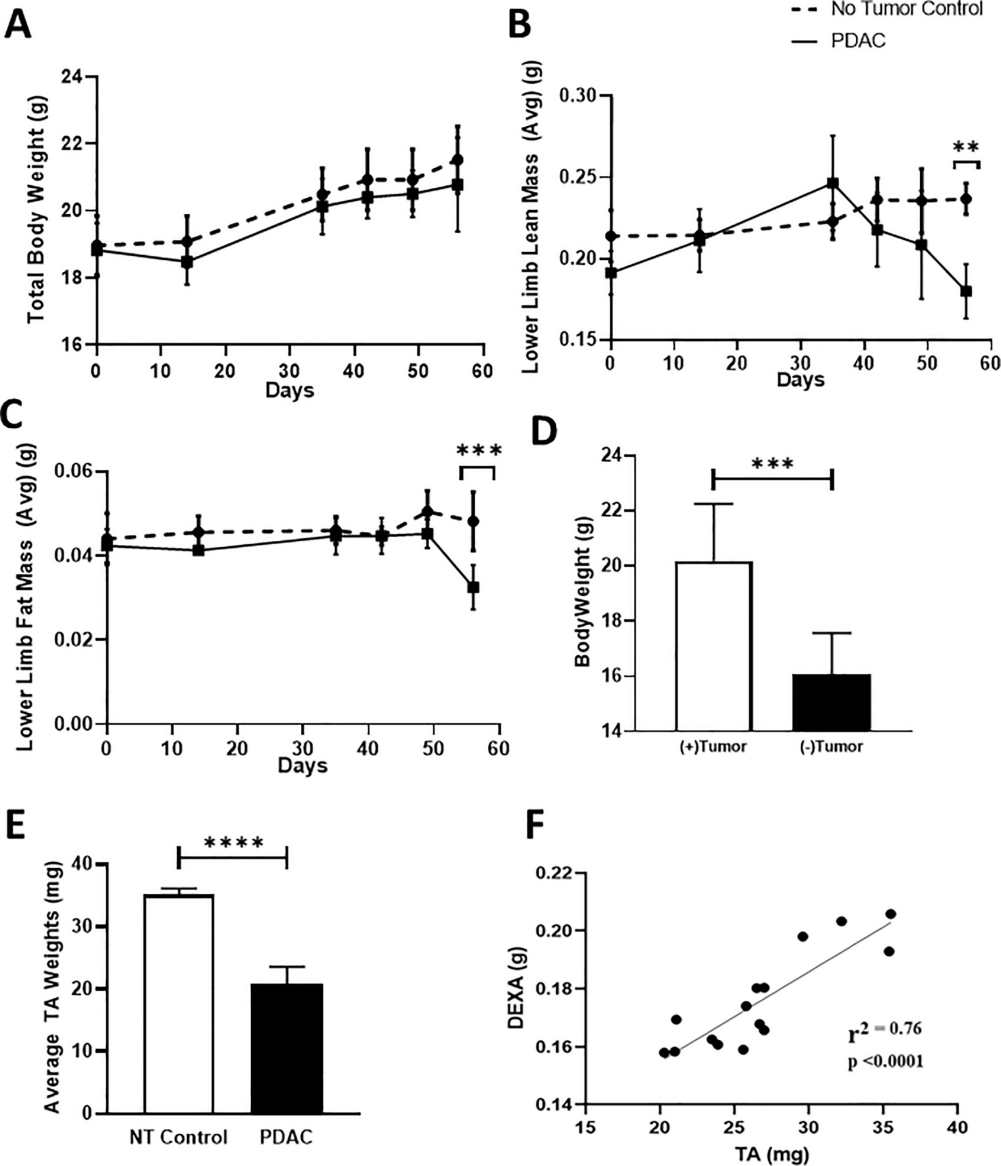
Longitudinal quantification of lean mass via DEXA demonstrates commencement of PDAC-related muscle wasting within 38 days of tumor implantation, which cannot be detected by assessment of total body weight. Mice were randomized to two groups prior to orthotopic tumor injections: 1) NTC; (n = 5) and 2) PDAC tumor bearing; (n = 5). Longitudinal DEXA and total body weight (TBW) measurements were performed as described in Materials and Methods. (A) Longitudinal TBW is presented for the NTC and PDAC mice. Longitudinal lower limb (B) lean and (C) fat mass was determined by DEXA. Data is presented as mean ± SD by two-way ANOVA for repeated measures (the interaction between PDAC vs. NTC and time was significant at day 56 for lean mass and fat mass **p = 0.002 and ***p = 0.0003, respectively), Tukey’s multiple comparison test. (D) The total body weight (TBW) (g) and (E) TA weights (mg) of the PDAC bearing mice was determined on the day of sacrifice, and the data are presented as TBW (+ tumor) before primary tumor harvest and net weight (- tumor) after primary tumor harvest for each mouse with the mean ± SD, Student t-test revealed a significant difference (***p = 0.0005) (****p<0.0001) between both bodyweight and TA weight, respectively. (F) A Pearson correlation analysis was performed on a separate and larger cohort of PDAC tumor bearing mice (n = 15) to determine the relationship between DEXA lean mass and TA weight. The analysis confirmed a significant (p < 0.0001) relationship between the two measures with r^2^ = 0.76. Data are expressed as mean ± SD.

To assess the onset and progression of SMW in PDAC-bearing mice, we performed longitudinal, in vivo DEXA analyses normalized to total body weight (TBW) (weight including the tumor), and compared the findings to those in no tumor controls (NTC). DEXA revealed a decrease in the lower limb mass of tumor-bearing mice beginning at day 38, resulting in a significant decrease vs. NTC mice at day 56 (mean ± SD: 0.18 ± 0.01 in PDAC mice vs. 0.23 ± 0.01 in NTC mice, p = 0.002) (Fig 3B), a decrease that could not be detected by the measurement of TBW (Fig 3A). The loss of lower limb lean muscle mass in the PDAC group was concomitant with a significant loss of lower limb fat mass during the same time period (mean ± SD: 0.03 ± 0.00 in PDAC mice vs. 0.05 ± 0.00 in NTC mice, p = 0.0003) (Fig 3C). Postmortem examination of each animal determined that TBW is an unreliable measure of the onset of SMW because the growing tumor mass compensates for the loss of viable tissue. Thus, the TBW of the tumor-bearing mice was significantly greater than their net weight (NW) (weight–tumor weight) (mean ± SD: 20.18 ± 2.071 for TBW vs. 16.09 ± 1.48 for NW, p = 0.0005) (Fig 3D), and this increase was commensurate with primary tumor weight, such that there were no differences in TBW between the PDAC and NTC groups. Our finding of significantly reduced TA muscle size, as determined by weight, in the tumor-bearing mice when compared to the NTC group is further evidence of PDAC-associated SMW (mean ± SD: 0.020 ± 0.002 in PDAC mice vs. 0.035 ± 0.009 in NTC mice, p<0.0001) (Fig 3E). A Pearson correlation analysis, performed on a larger cohort of PDAC mice (n = 15), confirmed a strong relationship between DEXA lower limb lean mass and TA weight (r^2^ = 0.76; p<0.0001) (Fig 3F). Due to the degree of tumor burden in some mice, we sought to understand whether or not tumor size had an impact on the severity of SMW. To investigate this, we performed a Pearson correlation analysis of tumor size vs. DEXA lower limb lean mass, and the results failed to demonstrate a significant relationship between tumor size and lower limb lean mass (r^2^ = 0.04; p = 0.44)(data not shown).

### Immunohistochemistry confirms PDAC-related SMW

**Fig 4:**
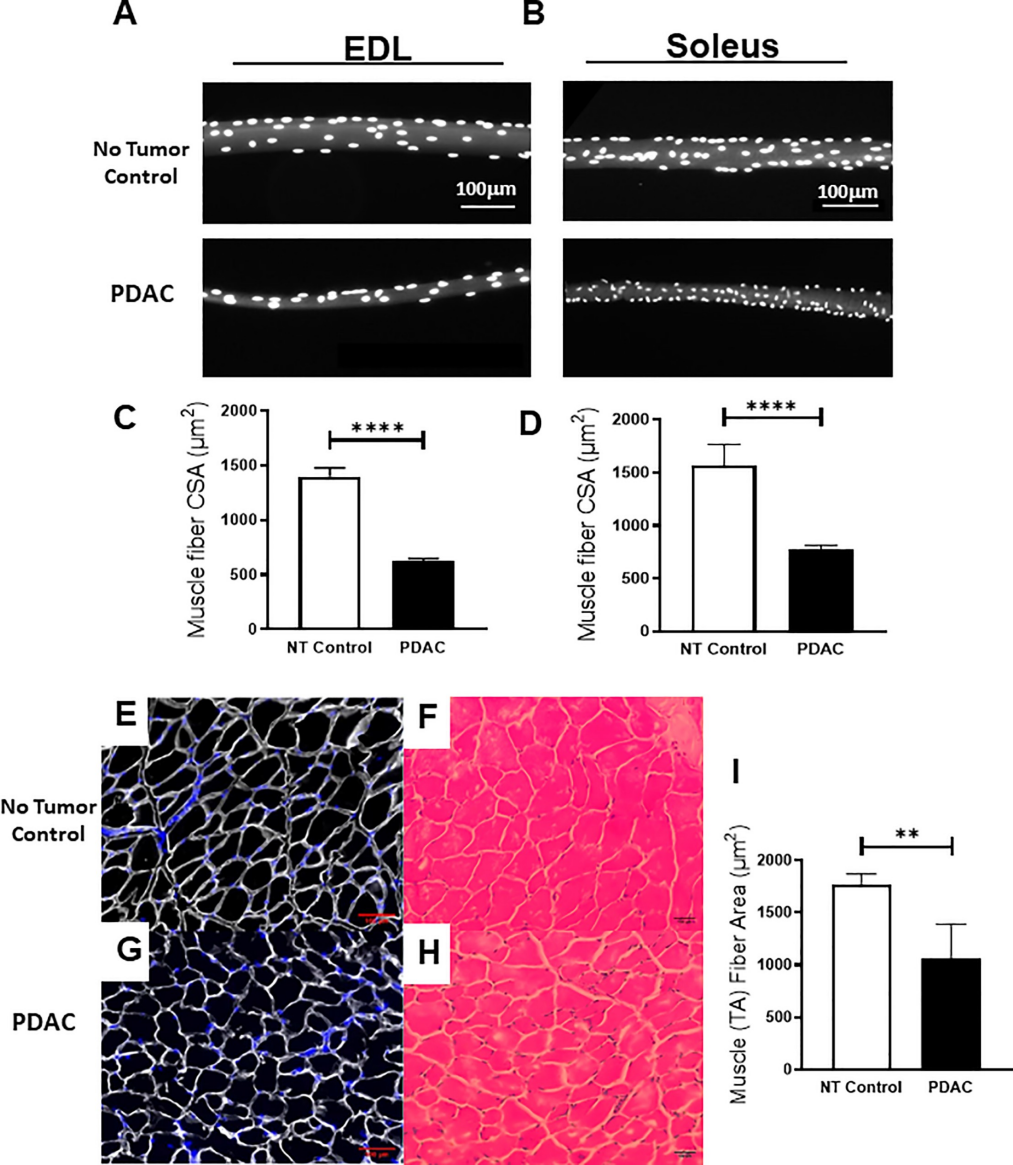
Histologic confirmation of skeletal muscle fiber atrophy in terminal mice with PDAC. Following euthanasia, the EDL and SOL muscles were harvested from PDAC baring and NTC mice and stained with DAPI for calculation of CSA (100 fibers per mouse). TA muscles were dissected and weighed. After which, TA muscles were frozen and stained with Laminin and H&E, and CSA was measured. Photomicrographs (10x) show representative DAPI stained (A) EDL and (B) SOL muscle fibers at the time of sacrifice from NT (n = 5) and PDAC baring mice (n = 5), with quantification of (C) EDL and (D) SOL CSA (a significant difference was found between NTC and PDAC mice, using Student’s t-test, mean ± SD ****p<0.0001). Images (10x) of laminin and H&E stained TA cross-sections of (E,F) NTC and (G,H) PDAC are shown, respectively, along with quantification of TA (I) CSA (μm^2^) (n = 5) and are presented with the mean ± SD (a significant difference was found between NTC and PDAC mice, using Student’s t-test, mean ± SD **p<0.01).

To confirm SMW in this model of PDAC, we performed histomorphometry on fast twitch (EDL) and slow twich (SOL) skeletal muscle fibers from the PDAC-bearing and NTC mice (Fig 4A and 4B). We found a significant decrease of 38 and 33% in the CSA of both EDL and SOL muscle fiber types, respectively, in PDAC mice. This finding is similar to reported decreases in mice with age and disease-related sarcopenia [30]. Histomorphemetry of TA sections (Fig 4E–4H) show an atrophied appearance, along with disorganized extracellular matrix. Quantification of the CSA of these muscle fibers (Fig 4I) substantiate SMW. Collectively, these results formally establish SMW with concomitant loss of fat mass in this PDAC model, which can be longitudinally assessed via DEXA scanning.

## Discussion

To better understand the mechanisms of PDAC-related SMW and identification of treatable targets, there remains a need for the development of small animal models with longitudinal outcomes. Thus, we utilized in vivo DEXA scans to predict lean muscle mass in a selected region of interest (ROI) of growing and PDAC mice. The lower portion of the hind limbs were selected as the ROI to omit the mass of the tumor from analysis. Using this method of analysis, we found strong, significant correlations between DEXA predicted lean mass and whole body and TA weight, in both the growing and PDAC mice. Further analysis, determined a strong relationship between DEXA predicted lower limb lean mass and whole body lean mass in growing mice. These findings suggest that DEXA can be used as a longitudinal outcome measure of PDAC-related SMW in a murine model. Due to its high reproducibility and accuracy of measurement of lean body mass, DEXA has emerged as one of the most promising longitudinal assessment tools for the direct measurement of lean mass and diagnosis of sarcopenia [16]. Currently, DEXA is widely used in clinical studies to diagnose sarcopenia, and is recognized as the gold standard [16]. NMR and micro-CT are techniques that have been used to measure total animal growth [17] and loss of body mass in models of cancer cachexia [22–24]. However, DEXA is considered a useful method for the determination of body composition, because it is simple, fast, noninvasive, and accurate [17]. Furthermore, it is highly accessible and less expensive than micro-CT and NMR and can measure all three tissues in the body, at specific regions. The reliability of DEXA to predict whole body composition with relatively minimal variation has been duly noted in the literature [17]. Like other noninvasive imaging techniques, it can reduce the number of animals needed per experiment and improve their quality of life. However, DEXA has some limitations when predicting lean mass in models of cancer cachexia. DEXA cannot specifically discern muscle quality, as does techniques such as micro-CT. Therefore, additional testing is needed to determine frailty or muscular performance. In addition, DEXA is sensitive to hydration status, which may become problematic when animals reach an advanced disease stage and may be incapable of accessing water ad libitum. Water may be placed on the floor of the cage to provide sick animals’ easier access. Additionally, water sources should be removed 1–2 hours before scanning to avoid over hydration. Furthermore, DEXA scans expose animals to radiation. Although the doses are small, researchers may have to take this into consideration when designing experiments in which the animal may be further compromised by radiation or if exposure to radiation will act as a confounder. Lastly, animals have to be anesthetized prior to DEXA scanning. Anesthetization of an already compromised animal may cause death. This is a reality that has to be consider in the experimental design.

Using a DEXA instrument for small animals to longitudinally quantify skeletal muscle mass in a cohort of PDAC mice (n = 5), we found a significant reduction in skeletal muscle and fat mass compared to their NTC littermates. This decrease in skeletal muscle and fat mass was concomitant with “failure to thrive,” and subsequent mandatory sacrifice of the animals. TBW was not different between groups at any of the time-points. Therefore, identification of the onset of SMW may be vital for the survival of animal models and patients with cancer, if it can be halted at that time. Clinical researchers agree that once muscle loss reaches a point of clinically obvious detriment, it is irreversible [31].

Indeed, the SMW and cachexia that are experienced by patients with cancer have been defined on a continuum that begins with pre-cachexia and ends with refractory cachexia, which unfortunately cannot be relieved [31, 32]. Therefore, it is important to understand when these disorders begin during disease progression and the mechanisms involved, when considering viable treatment options. To our knowledge, this is the first study to assess the validity of the syngeneic KCKO-luc cell orthotopic model of PDAC as a model of PDAC-associated SMW, and the utility of longitudinal DEXA analysis to assess SMW in tumor bearing mice. The longitudinal quantification of lean mass proved to be predictive of “failure to thrive,” which was an indication for euthanasia. Thus, the diagnosis and attenuation of PDAC-associated SMW in this model can ultimately translate to improving the quality of life and survival in patients with cancer.

In addition, patients who experience SMW have a lower tolerance for treatment and higher drug toxicity [13, 16, 33], which often results in treatment discontinuation. Treatment-associated toxicity in these patients may be the result of improper drug dosing regimens that are based on antiquated methodology [16]. Recent clinical evidence suggests the use of body composition measurement to improve drug dosing, reduce toxicity, and establish early intervention in patients with cancer [6, 34]. Therefore, we conclude that the use of DEXA as a tool to characterize SMW to improve dosing is an area that warrants further study. Recent research reports that SMW is independently prognostic of lower survival in patients with gastrointestinal cancers [35]. Martin et al. [36] show similar results in a large cohort of patients with cancer. This study concluded that survival in sarcopenic patients was 1/3 of their non-sarcopenic counterparts, regardless of body weight. In addition, increases in lean mass during NAT has been shown to be independently associated with progression to resection surgery in patients with PDAC [6], even when these patients experienced loss of adipose tissue. Notably, patients who experienced decreases in lean mass during or after NAT underwent surgical exploration, but not resection.

Not surprisingly, we found a significant difference in TBW of animals (with tumor), compared to the animals’ normalized weight without tumor. This finding suggests that TBW is a poor indicator of survival and general health due to the continuous growth of the tumor, which mask the loss of viable lean and fat mass in this animal model. These findings are significant because they specify that the longitudinal quantification of lean mass is a more appropriate prognostic indicator than the measure of TBW in this model of murine PDAC.

## Conclusion

We have demonstrated the efficacy of DEXA as a longitudinal outcome measure of PDAC-related SMW. PDAC is a disease with an extremely high mortality rate. Survival is further decreased by the onset of SMW. Early detection and treatment of SMW in affected patients may improve quality of life and survival. Therefore, more research is needed to understand the mechanism(s) that lead to a dysregulation in myocellular homeostasis. Research of this nature requires a pre-clinical model that provides longitudinal outcome measures of body composition and disease progression. In this study, we used DEXA as a longitudinal outcome measure to assess SMW in growing mice and in an established murine PDAC model. Utilizing this technique, we were able to detect the onset of SMW, which was commensurate with “failure to thrive” and was confirmed via TA analysis, body weight assessment, and histomorphometry. Considering conserved biological mechanisms of SMW, these results may be applicable to all tumor models associated with cachexia.

## Supporting information

S1 Data (XLSX)
